# Genotypic variation in Norway spruce correlates to fungal communities in vegetative buds

**DOI:** 10.1111/mec.15314

**Published:** 2019-12-09

**Authors:** Malin Elfstrand, Linghua Zhou, John Baison, Åke Olson, Karl Lundén, Bo Karlsson, Harry X. Wu, Jan Stenlid, M. Rosario García‐Gil

**Affiliations:** ^1^ Uppsala Biocentre Department of Forest Mycology and Plant Pathology Swedish University of Agricultural Sciences Uppsala Sweden; ^2^ Umeå Plant Science Centre Department of Forest Genetics and Plant Physiology Swedish University of Agricultural Sciences Umeå Sweden; ^3^ Skogforsk Svalöv Sweden

**Keywords:** cherry spruce rust, peroxidase, phenology, *Picea abies*, *Rhizosphaera kalkhoffii*, *Sydowia polyspora*

## Abstract

The taxonomically diverse phyllosphere fungi inhabit leaves of plants. Thus, apart from the fungi's dispersal capacities and environmental factors, the assembly of the phyllosphere community associated with a given host plant depends on factors encoded by the host's genome. The host genetic factors and their influence on the assembly of phyllosphere communities under natural conditions are poorly understood, especially in trees. Recent work indicates that Norway spruce (*Picea abies*) vegetative buds harbour active fungal communities, but these are hitherto largely uncharacterized. This study combines internal transcribed spacer sequencing of the fungal communities associated with dormant vegetative buds with a genome‐wide association study (GWAS) in 478 unrelated Norway spruce trees. The aim was to detect host loci associated with variation in the fungal communities across the population, and to identify loci correlating with the presence of specific, latent, pathogens. The fungal communities were dominated by known Norway spruce phyllosphere endophytes and pathogens. We identified six quantitative trait loci (QTLs) associated with the relative abundance of the dominating taxa (i.e., top 1% most abundant taxa). Three additional QTLs associated with colonization by the spruce needle cast pathogen *Lirula macrospora* or the cherry spruce rust (*Thekopsora areolata*) in asymptomatic tissues were detected. The identification of the nine QTLs shows that the genetic variation in Norway spruce influences the fungal community in dormant buds and that mechanisms underlying the assembly of the communities and the colonization of latent pathogens in trees may be uncovered by combining molecular identification of fungi with GWAS.

## 1 INTRODUCTION

At any given time during their life cycle, trees are colonized by a wide range of microbes, including fungi. These fungi may reside both epiphytically, on the surface of the tissue, and endophytically, within the host tissue without causing any visible symptoms (Arnold, Henk, Eells, Lutzoni, & Vilgalys, 2007; Rajala et al., 2013; Rodriguez, White, Arnold, & Redman, 2009; Sieber, 2007). The epi‐ or endophytic fungi associated with a tree have varying ecological roles, such as being mutualistic symbionts (latent) pathogens or facultative saprotrophs (Rajala, Velmala, Vesala, Smolander, & Pennanen, 2014; Saikkonen, 2007). Pathogenic fungi cause damage to the host that can reduce host growth and fitness, such as by decreasing photosynthetic capacity, and causing premature leaf shed or lesion formation (Hanso & Drenkhan, 2012; Pan et al., 2018). Highly diverse fungal communities have been reported in studies on current‐year Norway spruce needles, that is those that flushed during the sampling season (Menkis, Marciulynas, Gedminas, Lynikiene, & Povilaitiene, 2015; Nguyen, Boberg, Ihrmark, Stenström, & Stenlid, 2016). It has been shown that the needle endophyte community in Norway spruce (*Picea abies* L., Karst) varies among genotypes (Rajala et al., 2013, 2014). This would indicate that apart from the presence of fungi with the capacity to colonize the host, environmental and climatic factors (Eusemann et al., 2016; Menkis et al., 2015; Millberg, Boberg, & Stenlid, 2015; Moler & Aho, 2018; Nguyen et al., 2016; Rodriguez et al., 2009), the assembly of the phyllosphere community associated with a given tree will also depend on factors encoded by the host's genotype (Cordier, Robin, Capdevielle, Desprez‐Loustau, & Vacher, 2012; Rajala et al., 2013, 2014). Although it is hypothesized that the plant‐inhabiting community is synergistically determined by environmental and host genetic factors (Horton et al., 2014; Rajala et al., 2013; Terhonen, Blumenstein, Kovalchuk, & Asiegbu, 2019), the role of specific host genetic variation under natural conditions is poorly understood. Reports on specific host genetic variation that affects the phyllosphere community composition come from annual plant species and deal primarily with bacterial colonization (Horton et al., 2014; Roman‐Reyna et al., 2019; Wallace, Kremling, Kovar, & Buckler, 2018). In a study of 196 accessions of *Arabidopsis thaliana*, Horton et al. (2014) showed that both the presence/absence and abundance of fungal species in the associated phyllosphere communities are influenced by the *Arabidopsis* genotype, but for only the more abundant species in the study. Genome wide association study (GWAS) results have implicated a role of both defence responses and cell‐wall integrity in the assembly of *Arabidopsis* phyllosphere fungal communities (Horton et al., 2014). However, hitherto there have been no reports on specific genetic variants affecting phyllosphere fungal community composition in perennial plant species, such as trees. These have a more complex architecture (relative to most annual plants) and are exposed to their changing biotic and abiotic environment over many consecutive growth cycles. These interactions between genetics, environment and time are likely to significantly influence the structure of the phyllosphere community of a host tree.

Conifers dominate the boreal forests in the Northern Hemisphere (Farjon & Page, 1999). Owing to their often large population sizes, outbreeding mating systems and efficient gene flow (wind pollination), conifers are characterized by high levels of heterozygosity and intraspecific diversity, which is reflected in low levels of genetic differentiation between populations (Savolainen, Pyhäjärvi, & Knürr, 2007). Norway spruce is one of the most important conifer species in Europe, both ecologically and economically. Together with Scots pine (*Pinus sylvestris* L.), it essentially makes up the continuous boreal forests of the continent. The fungal community composition of Norway spruce needles has been reported to change along a latitudinal gradient on a continental scale as well as between individual genotypes of Norway spruce within a stand (Nguyen et al., 2016; Rajala et al., 2013, 2014), a pattern which may be considered consistent with horizontal transfer of the fungi, and possibly also with the high intraspecific diversity in conifers (Prunier, Verta, & MacKay, 2016).

The development of high‐throughput sequencing (HTS) methods paved the way for the generation of the first draft assembly of the Norway spruce genome (Nystedt et al., 2013). The availability of the genome sequence for Norway spruce has opened new possibilities for the development of genetic markers to produce highly resolved genotypes of an individual tree (Vidalis et al., 2018) and to conduct GWAS (Baison et al., 2019). Similarly, sequencing of the ITS (internal transcribed spacer) region with HTS methods has allowed mycologists to describe fungal communities and community dynamics in various ecosystems in greater detail than before, advancing functional understanding of various ecological processes and phylogenetic relationships (Clemmensen et al., 2013; Kubartová, Ottosson, Dahlberg, & Stenlid, 2012; Rosling et al., 2011; Seena & Monroy, 2016; Tedersoo et al., 2014; Voříšková & Baldrian, 2012). The combination of ITS sequencing of phyllosphere fungi with the recently available genotyping resources in Norway spruce in an association study may provide insights in to how tree phyllosphere communities assemble, through the identification of specific fungal taxa and with genetic variants associated with general shifts, in the tree phyllosphere communities. For instance, the communities of seemingly healthy needles often include known (e.g., *Lirula macrospora* or *Rhizosphaera kalkhoffii*) or suspected (e.g., *Phoma herbarum* and *Sydowia polyspora*) needle pathogens (Menkis et al., 2015; Nguyen et al., 2016; Rajala et al., 2013, 2014). Furthermore, in a recent metatranscriptomics study of Norway spruce tissues, similar frequencies of fungal transcripts were found both in needle and in bud samples (Delhomme et al., 2015), suggesting that vegetative buds as well as needles harbour active fungal communities, but the study provided no insights into the composition of the bud community.

Here we report the results of a study in the perennial conifer Norway spruce that combined ITS sequencing of the fungal communities associated with dormant buds with GWAS of 478 individuals to (a) describe the fungal community associated with dormant buds in a large population of unrelated trees and thus describe the abundance of possible latent pathogen colonizations of asymptomatic tissues, (b) suggest loci in the genome of Norway spruce that correlate with variation in the fungal communities across the studied population, and (c) identify loci in the genome of Norway spruce that correlate with the presence of specific, latent, pathogens.

## 2 MATERIALS AND METHODS

### 2.1 Amplification, sequencing and analysis of phyllosphere fungal community

Healthy looking vegetative buds were sampled from 518 trees in the southern Swedish Norway spruce breeding archives, located at Ekebo and Maltesholm. The trees in the breeding archive were planted approximately 35 years ago with 7 m between each tree and 5 m between rows. Buds were collected by hand from exposed branches about 2 m above ground. The collected buds were placed into labelled Ziploc plastic bags. In the field, the samples were stored in sterox boxes filled with cooling blocks. After each day of field work the samples were transferred to –20°C for long‐term storage. Total genomic DNA was extracted from approximately five buds per tree using the Qiagen Plant DNA extraction kit (extraction details as described by Baison et al., 2019).

DNA samples were amplified separately using the primer pair gITS7 (5′‐xxxxxxxxGTGARTCATCGARTCTTTG‐3′) and ITS4 (5′‐xxxxxxxxTCCTCCGCTTATTGATATGC‐3′) (Ihrmark et al., 2012) containing 8‐bp sample identification barcodes denoted by x, as previously described (Clemmensen, Ihrmark, Durling, & Lindahl, 2016). Each sample was amplified using unique barcode combinations. The gITS7–ITS4 amplicon amplifies the ITS2 region, which provides good species resolution capacity and can be sequenced throughout the entire length with available HTS technologies (Clemmensen et al., 2016; Ihrmark et al., 2012).

Prior to the amplification of the ITS2 region, DNA quantification was performed using the Qubit ds DNA Broad Range Assay Kit (ThermoFischer) and samples were diluted when needed ensuring sample concentration in the range of 1–10 ng/µl. PCR amplifications were done according to Clemmensen et al. (2016). In brief 50‐µl reactions with a final concentration of 1× DreamTaq Green Buffer, 200 µm dNTPs, 750 µm MgCl_2_, 1.25 µl DreamTaq polymerase (ThermoFischer Scientific), 0.3 µm Tagged gITS7/ITS4 primer mix and approximately 2.5 ng of template DNA were run with the following cycle conditions: 5 min at 94°C, 27 cycles of 30 s at 94°C, 30 s at 56°C and 30 s 72°C, and a final 7 min at 72°C; blanks were run as negative controls. The resulting amplicons were purified with the AMPure kit (Beckman Coulter). The concentration of purified PCR products was determined fluorometrically using the Qubit ds DNA Broad Range Assay Kit (ThermoFischer), and equimolar mixes of PCR products from 88 samples, including the blanks, were pooled, creating six sample pools that were used for PacBio SMRT circular consensus sequencing using eight SMRT cells per pool. Construction of the sequencing library and sequencing were carried out by NGI SciLifeLab.

The sequences generated were subjected to quality control and clustering in the scata NGS sequencing pipeline (http://scata.mykopat.slu.se). Quality filtering of the sequences included the removal of short sequences (<200 bp), sequences with low read quality (any base in the sequence that has a PHRED score < 10) and primer dimers; homopolymers were collapsed to 3 bp before clustering. Sequences that were missing a tag or primer were excluded. The primer and sample tags were then removed from the sequence, but information on the sequence association with the sample was stored as metadata. The sequences were then clustered into different operational taxonomic units (OTUs) that essentially correspond to the species level by single‐linkage clustering based on 98.5% similarity. The most common genotype (real read) for clusters was used to represent each taxon. For clusters containing two sequences, a consensus sequence was produced. The fungal taxa were taxonomically identified using the UNITE database version 7.2 (https://unite.ut.ee/index.php) and the blastn algorithm. The criteria used for identification were as follows: sequence coverage > 80%, similarity to species level 98%–100% and similarity to genus level 94%–97%. Sequences not matching these criteria were considered unidentified. To obtain further information on abundant yet unidentified sequences (i.e., suspected Norway spruce clusters), a secondary search was performed in GenBank (NCBI) using the blastn algorithm. After removal of singletons and nonfungal sequence reads, the relative abundances of each fungal cluster (OTU) in each sample were calculated. The data set was tested for PCR (polymerase chain recation) contamination by analysing the PCR blanks, and confounding factors such as variation between sequencing libraries, sites and site characteristics. From the original population samples, 473 trees growing at the Maltesholm site were selected for subsequent analysis.

The 1% most abundantly sequenced OTUs were analysed with multivariate ordination methods in past 3.20 (Hammer, Harper, & Ryan, 2001). To identify the main drivers in the data set, a principal component analysis (PCA) was made and the first six eigenvectors from the PCA were analysed in the subsequent GWAS.

OTUs including more than 2% of the reads, corresponding to known conifer pathogens and with a presence in at least 35% of the Norway spruce samples, were selected for targeted GWAS of latent pathogens (Figure S3). Separate files with either relative abundance or presence/absence data were prepared for each of the OTUs that met the criteria for GWAS of latent pathogens and were used in trait‐association mapping.

### 2.2 Norway spruce genotyping and SNP annotation

Generation and evaluation of Norway spruce exome capture is described elsewhere (Vidalis et al., 2018). In brief, 478 samples from a subset of 9,000 maternal trees on which sequence capture was performed using 40,018 previously evaluated diploid probes and samples, were sequenced to an average depth of 15×. Illumina sequencing compatible libraries were amplified with 14 cycles of PCR with the probes being hybridized to a pool comprising 500 ng of eight equimolarly combined libraries following Agilent's SureSelect Target Enrichment System (Agilent Technologies) protocol. These enriched libraries were then sequenced on an Illumina HiSeq 2500 using the 2 × 100‐bp sequencing mode.

Read mapping and initial variant calling is described in detail by Baison et al. (2019). Basically, the sequence reads were aligned to the Norway spruce genome using the Burrows–Wheeler Aligner (BWA; Li & Durbin, 2010) and variant detection utilized the Genome Analysis Software Kit (gatk; DePristo et al., 2011; McKenna et al., 2010) best practice pipeline with a training subset created for application in the Variant Quality Score Recalibration (VQSR) method. Only bi‐allelic single nucleotide polymorphisms (SNPs) with a minor allele frequency (MAF) and “missingness” of <0.05 and >20%, respectively, were removed. For the selected set of trees a total of 178,101 SNPs passed the filtering and were used for downstream analysis. Annotation was performed using default parameters of snpeff 4 (Cingolani et al., 2012) and local Norway spruce genome annotated database. Ensembl general feature format (GTF, gene sets) information was utilized to build the *Picea abies* snpeff database.

### 2.3 Trait association mapping

Loadings on the first six axes from the PCA of the relative abundance data of the 1% largest OTUs were used for the GWAS, with each individual axis explaining at least 5% of the variance in the detected phyllosphere communities. Subsequently, the relative abundance or presence/absence data for the OTUs (OTU_5, OTU_9, OTU_15 and OTU_19) on each host genotype were used for the trait‐association mapping.

The statistical LASSO model as described by Li et al. (2014) was applied to the traits associated with the detected phyllosphere communities and phyllosphere pathogens.

The LASSO model is:

$$\min_{\left(\alpha_{0},\alpha_{j}\right)}\frac{1}{2n}\sum_{i=1}^{n}{\left(y_{i}-\alpha_{0}-\sum_{j=1}^{p}x_{\mathrm{ij}}\alpha_{j}\right)}^{2}+\lambda \sum_{j=1}^{p}\left|\alpha_{j}\right|,$$

where *y_i_* is the phenotypic value of an individual *i* (*i* = 1, …, *n*; *n* is the total number of individuals), *α*_0_ is the population mean parameter, *x_ij_* is the genotypic value of individual *i* and marker *j* coded as 0, 1 and 2 for three marker genotypes AA, AB and BB, respectively, *α_j_* is the effect of marker *j* (*i* = 1, …, *n*; *n* is the total number of markers), and *λ* (>0) is a shrinkage tuning parameter. A fundamental idea of LASSO is to utilize the penalty function to shrink the SNP effects toward zero, and only keep a small number of important SNPs which are highly associated with the trait in the model.

The stability selection probability (SSP) of each SNP being selected by the model was applied to determine significant SNPs (Gao et al., 2014; Li & Sillanpää, 2015). For a marker to be declared significant, an SSP inclusion ratio (Frequency) was calculated for all selected SNPs for each trait and a minimum inclusion frequency of 0.56 was chosen as the most prudent cut‐off. The set of markers with nonzero effects was recorded; after bootstrapping, this provides an approximation of a *p*‐value. The SSP threshold used for defining significant SNPs was estimated as suggested by Meinshausen and Bühlmann (2010). The threshold is calculated based on the number of SNPs, number of individuals included in the subset and expected number of false positives. A lambda of 250 with 1,000 bootstraps and a false positive cutoff of five (5) were applied to the entire association analysis. Population structure was accounted for in all analyses by including the first five components from a PCA of the genotypic data as covariates in the LASSO model and the total variance explained by the five PCs was 19.3%. Finally, an adaptive LASSO approach (Zou, 2006) was used to determine the percentage of phenotypic variance (PVE; *H* ^2^_QT_) of all quantitative trait loci (QTLs). The analyses were all performed with *glmnet* in rstudio, R version 3.4.0 (Team, 2015), and the codes used can be found at https://github.com/RosarioGarciaLab/Norway-Spruce-Association-Mapping.

Information on putative candidate genes associated with the QTLs and the expression pattern of the candidate genes in the Norway spruce clone Z4006 were collected from the publicly available Norway spruce genome portal and *P. abies* exAtlas (https://www.congenie.org). The position of the detected QTLs in the Norway spruce genome was estimated by searching an ultradense genetic map (Bernhardsson et al., 2019) for markers derived from the same probes from which the SNP markers holding the QTLs originated.

## 3 RESULTS

### 3.1 Norway spruce buds are colonized by well‐known phyllosphere endophytes and pathogens

After quality control, 676,375 ITS sequence reads remained. At 98.5% similarity, these sequences clustered into 4,899 OTUs, excluding 3,357 singletons. Fungal taxa were identified using the UNITE database. However, the dominant cluster (85% of reads, a spruce ITS sequence [AJ243167] and 10 other nonfungal clusters were removed from the data set leaving 4,888 OTUs [104,768 sequence reads]). After removal of the plant clusters the median read count over the samples in the data set was 148 reads. Relative abundances in each sample were calculated on the remaining OTUs. After testing and removal of confounding factors and of samples with poor amplification/sequencing success (total number of remaining fungal reads <20) the analyses were limited to the largest clusters only (Horton et al., 2014), leaving 473 Norway spruce samples and 49 OTUs (top 1%, including 80% of the fungal reads, Supporting Information S1) in the subsequent analyses. The majority of these belonged to Ascomycota, 56% belonged to Pezizomycotina alone and two OTUs (4%) represented Taphrinomycotina (Figure S2). The basidiomycete orders Agaricomycotina (25%), Pucciniomycotina (13%) and Ustilagomycotina (2%) were among the most abundant OTUs (Figure S2). Several of the most abundant OTUs represented yeasts (e.g., OTU_2 and OTU_1), and correspond to the basidiomycete yeasts *Curvibasidium cygneicollum* and *Filobasidium wieringae,* respectively while OTU_6 corresponds to the common yeast‐like ascomycete *Aureobasidium pullulans* (Table 1). The abundant OTU_3 is probably a member of the *Cladosporium herbarum* complex (Table 1). Other OTUs comprise well‐known needle endophytes, such as *Ceramothyrium* (OTU_11) and *Perusta inaequalis* (OTU_17) (Table 1). OTU_4, one of the most abundant OTUs, is apparently a hitherto undescribed ascomycete fungus (Table 1), and is identical to the relatively abundant sequence cluster “Unidentified sp. 2168_9” reported from the Norway spruce phyllosphere (Menkis et al., 2015). Several of the abundant OTUs represent needle pathogens: OTU_9 (*Thekopsora areolata*), OTU_15 (*Rhizosphaera kalkhoffii*) and OTU_19 (*Lirula macrospora*), or latent pathogens: OTU_5 (*Sydowia polyspora*), OTU_24 (*Botrytis cinerea*) and OTU_30 (*Phoma herbarum*) (Table 1).

**Table 1:** Putative taxonomic assignment for the most abundant operational taxonomic units (OTUs)

Cluster ID^a^	Cluster size^b^	Samples (%)^c^	Reference^d^	Accession^d^	Score	E‐value	%
OTU_2	10,385	81.5	*Curvibasidium cygneicollum*	KY102972	573	3.00E‐162	100
OTU_1	9,573	78.3	*Filobasidium wieringae*	KY103446	608	8.00E‐173	100
OTU_4	8,116	85.6	Fungi	KP897174	460	2.00E‐128	100
OTU_3	6,942	96.1	*Cladosporium herbarum*	MF326605	449	4.00E‐125	100
OTU_5	6,855	81.7	*Sydowia polyspora*	KU837235	473	2.00E‐132	100
OTU_6	5,878	81.3	*Aureobasidium pullulans*	MF687197	460	2.00E‐128	100
OTU_8	4,796	76.5	*Vishniacozyma victoriae*	MF927673	433	4.00E‐120	100
OTU_10	4,018	42.6	Fungi	KP897236	473	2.00E‐132	100
OTU_7	3,716	71.6	*Itersonilia pannonica*	KY103613	470	3.00E‐131	100
OTU_11	2,604	49.5	*Ceramothyrium* sp.	KC978733	427	2.00E‐118	97.3
OTU_12	2,337	61.9	Xylariales	KP897169	494	2.00E‐138	100
OTU_17	2,259	63.9	*Perusta inaequalis*	AY560007	473	2.00E‐132	100
OTU_19	2,200	39.8	*Lirula macrospora*	HQ902159	392	6.00E‐108	99.1
OTU_9	2,183	41.4	*Thekopsora areolata*	DQ087231	575	8.00E‐163	100
OTU_14	2,178	64.1	*Tristratiperidium* sp.	KT696538	451	1.00E‐125	97.0
OTU_15	2,057	57.6	*Rhizosphaera kalkhoffii*	KY003236	475	6.00E‐133	100
OTU_18	1,813	77.5	*Sporobolomyces roseus*	MF102879	555	9.00E‐157	100
OTU_13	1,581	71.0	Melampsoraceae	KC883505	593	2.00E‐168	99.7
OTU_24	1,190	72.8	*Botrytis cinerea*	MF996364	444	2.00E‐123	100
OTU_23	1,084	65.9	*Ramularia vacciniicola*	KY979777	435	1.00E‐120	100
OTU_20	995	60.0	*Rhodosporidiobolus colostri*	KY104695	556	3.00E‐157	100
OTU_22	908	60.0	*Exobasidium* sp.	EU692770	366	4.00E‐100	96.4
OTU_25	824	60.4	Cystofilobasidiales	HM240834	571	1.00E‐161	100
OTU_37	807	61.1	Leotiomycetes	KM519282	433	4.00E‐120	99.2
OTU_29	785	64.5	*Taphrina carpini*	AY239215	536	3.00E‐151	99.7
OTU_21	745	53.8	Fungi	KP891308	654	0	100
OTU_30	726	65.5	*Phoma herbarum*	KT355016	460	2.00E‐128	100
OTU_34	690	56.0	Helotiales	AY971722	377	2.00E‐103	99.5
OTU_36	647	56.6	*Dothiora* sp.	KU728514	396	5.00E‐109	94.3
OTU_39	584	54.6	*Cyphellophora sessilis*	KP400571	518	1.00E‐145	100
OTU_43	547	55.8	Fungi	KP897199	442	6.00E‐123	100
OTU_44	539	58.2	Dothideomycetes	FR773250	433	4.00E‐120	100
OTU_41	459	47.7	*Phaffia* sp.	HF558647	590	3.00E‐167	97.2
OTU_45	432	48.3	*Trichomerium* sp.	KP004468	444	2.00E‐123	97.0
OTU_50	408	48.5	*Bannozyma* sp.	KY558343	551	3.00E‐156	99.7
OTU_53	383	53.8	*Cyphellophora*	AM902002	484	1.00E‐135	100
OTU_49	375	50.7	Chaetothyriales	GQ999538	518	1.00E‐145	99.7
OTU_48	369	43.4	*Phallus impudicus*	UDB015413	536	3.00E‐151	100
OTU_55	366	51.9	Fungi	KP897431	529	5.00E‐149	100
OTU_56	362	52.3	Botryosphaeriales	KM216350	427	2.00E‐118	99.2
OTU_57	355	55.4	*Dioszegia aurantiaca*	KY103346	407	2.00E‐112	100
OTU_54	353	52.1	*Genolevuria* sp.	KF036585	460	2.00E‐128	97.4
OTU_47	348	45.6	*Cystofilobasidium capitatum*	KY103158	593	2.00E‐168	100
OTU_72	301	21.6	*Naevala* sp.	JN120378	429	5.00E‐119	98.8
OTU_61	300	23.0	*Alternaria infectoria*	Y17066	468	1.00E‐130	100
OTU_62	292	18.6	*Taphrinia padi*	KU134843	564	2.00E‐159	99.7
OTU_60	286	18.8	Fungi	KP897203	477	2.00E‐133	100
OTU_77	267	23.0	*Symmetrospora* sp.	EU002876	507	3.00E‐142	97.0

a Cluster_ID is the identity of the assembled OTU.

b Cluster size is the number of reads that are associated with the OTU.

c Samples (%) is the frequency of the OTU presence in the 493 samples.

d Thetaxonomic assignment where “Reference” is the taxonomic assignment based on blastn searches in UNITE,”Accession” is the best hit, “score” and “E‐value” are the blast score and E‐values, and “%” is thepercentage identity in the alignment.

**Figure 1:**
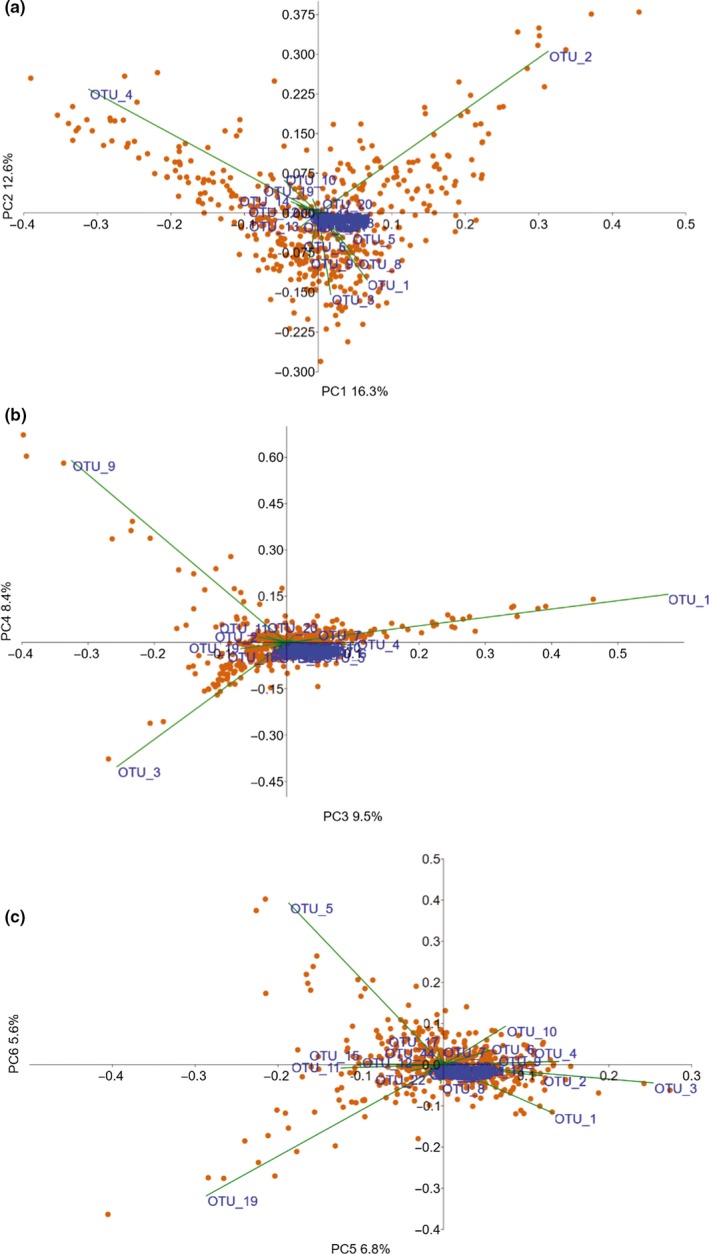
Principal component analysis of the operational taxonomic unit (OTU)/species abundance data. Biplots of the PCA on the abundance data of the 1% most heavily sequenced OTUs, used for the GWAS analysis. Orange symbols represent the individual samples. Green lines with blue OTU names represent the OTU loadings on the axis: (a) PC1 and PC2, (b) PC3 and PC4, and (c) PC5 and PC6

To reduce the number of variables for the subsequent GWAS analysis, the relative abundance data of the OTUs/species with the highest number of reads were used in a PCA to identify the most prominent drivers in the data set. The first two principal components (PCs) explained 14.9% and 12.8% of the total variance respectively in the phyllosphere community. The first and second PCs were strongly influenced by the abundance data of the undescribed ascomycete (OTU_4) and *C. cygneicollum*, while the second axis was also driven by *C. herbarum*, *S. polyspora* and OTU_10 (unclassified fungus; Figure 1a; Table 1). In addition to several of the taxa shaping the first two PCs, *F. wieringae* appeared to be an important driver on the third, and *T. areolata* on the fourth (Figure 1b; Table 1). PCs 5 and 6 explained 7.8% and 6.3% of the variance, respectively but the axes were shaped by mostly different OTUs than the first axis, such as *A. pullulans*, *T. areolata*, *R. kalkhoffii*, *Ceramothyrium* sp. and *L. macrospora* (Figure 1c; Table 1).

### 3.2 Trait association‐mapping

**Figure 2:**
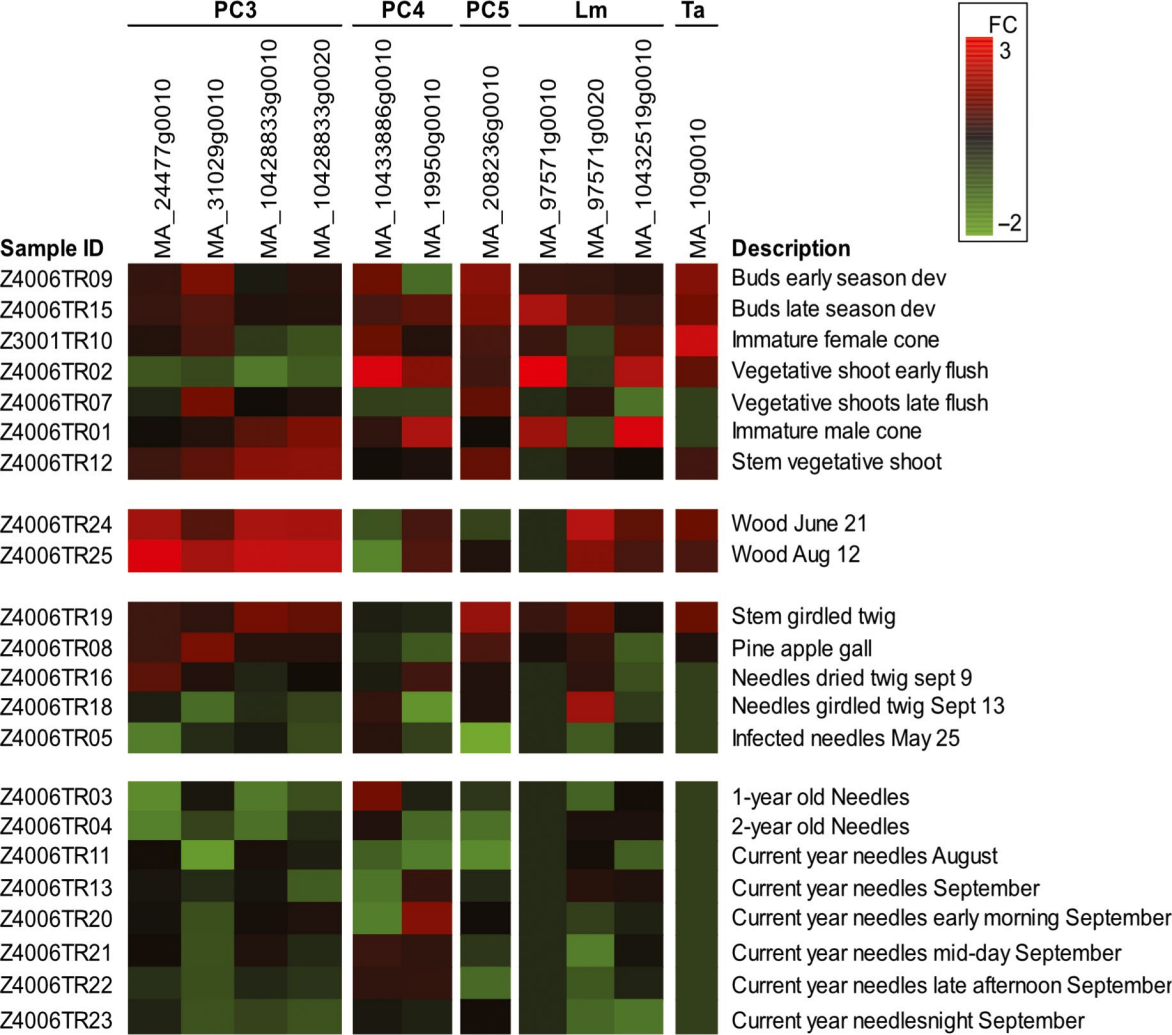
Expression of candidate genes in the *Picea abies* exAtlas. The heatmap depicts the relative expression levels (log_2_ fold change [FC]) of the candidate genes in select samples collected from the *Picea abies* exAtlas (https://www.congenie.org). The heatmap shows high (upregulated) relative expression in red colours and low (downregulated) relative expression in green as indicated in the bar at the bottom

Using the loadings on the first six PCs of the PCA (Figure 1) for GWAS, six QTLs associated with the fungal community that determine the PCs were detected (Table 2). The contigs of four of these QTLs could be identified on different linkage groups in an ultradense Norway spruce genetic linkage map (Bernhardsson et al., 2019; Table S4), indicating that the QTLs are located at different positions in the genome. The three SNPs that were significantly associated with PC3 are positioned in or adjacent to four Norway spruce gene models: MA_24477g0010, MA_31029g0010, MA_10428833g0010 and MA_10428833g0020 (Table 3). The gene ontology (GO) terms GO:0007264 (small GTPase mediated signal transduction) and GO:0006886 (intracellular protein transport) are associated with the gene model MA_24477g0010 that is similar to the *Arabidopsis* ADP‐ribosylation factor A1F gene (Table 3). MA_10428833g0020, encoding a putative ubiquitin‐protein ligase, is associated with the terms GO:0006817 (phosphate ion transport), GO:0016036 (cellular response to phosphate starvation), GO:0010337 (regulation of salicylic acid metabolic process), GO:0080021 (response to benzoic acid) and GO:0009626 (plant‐type hypersensitive response). Neither of the other two gene models have any functional information connected to them in the Norway spruce genome portal. However, MA_31029g0010 is homologous to AT3G59340, a putative solute carrier family 35 protein which is reported to be located in *Arabidopsis* chloroplasts. Inspection of the expression patterns of MA_24477g0010, MA_31029g0010, MA_10428833g0010 and MA_10428833g0020 reported in the *Picea abies* exAtlas (Nystedt et al., 2013) suggest that the candidate genes may be highly expressed in bark phloem, cambium and primary xylem (samples Z4006TR12, Z4006TR24, Z4006TR25 and Z4006TR19) and that relatively high levels of MA_31029g0010 transcript accumulate in vegetative shoots, developing buds, pine apple galls and immature female cones (Figure 2). Interestingly, the gene model MA_208236g0010 associating with PC5 showed an expression pattern reminiscent of MA_31029g0010 with relatively high transcript expression accumulating in vegetative shoots and developing buds (Figure 2). There is no functional information for MA_208236g0010 in the Norway spruce genome portal but the putative *Arabidopsis* orthologue AT2G47820 encodes an arginine‐glutamic acid dipeptide repeat protein, and PLAZA Homology enrichment analysis (https://bioinformatics.psb.ugent.be/plaza/) suggests that the molecular function of GO:0003677 (DNA binding) is associated with the product of the gene model.

**Table 2:** Significant association in the GWA study

Trait^a^	SNP^b^	Allele	SNP feature^c^	Frequency^d^	PVE^e^
PC3	MA_24477_24501	T*C	downstream_gene	0.649	
	MA_31029 _9337	C*G	missense	0.633	
	MA_10428833_21190	C*T	upstream_gene	0.552	
PC4	MA_19950_16139	G*C	upstream_gene	0.533	
	MA_10433886_12255	T*A	upstream_gene	0.682	1.2%
PC5	MA_208236_3389	A*G	stop_retained	0.565	
*Lirula macrospora*	MA_97571_20468	C*A	upstream_gene	0.566	
	MA_10432519_8378	C*A	synonymous	0.566	
*Thekopsora areolata*	MA_10_25927	G*A	upstream_gene	0.993	

a The trait upon which the marker associates, PC3–PC5 indicate the associations with loadings on the respective PC, and *L. macrospora* and *T. areolata* specify associations with the presence/absence data of these fungi among the samples.

b The SNP name consists of the contig (MA_number) and SNP position on the contig. For example, the first SNP MA_24477_24501 was located on contig MA_24477 at position 24,501 bp.

c Allelic variation associated with the SNP.

d Stability selection probability inclusion ratios for markers declared significant.

e Phenotypic variance explained (only values larger than 1.0% are displayed).

**Table 3:** Candidate Norway spruce gene models associated with the community composition and pathogen presence

Trait^a^	Candidate gene	GO terms^b^	Orthologues^c^	Best BlastX hit^d^
PC3	MA_24477g0010	GO:0005215—transporter activity, GO:0005525—GTP binding, GO:0005794—Golgi apparatus, GO:0006471—protein ADP‐ribosylation, GO:0006886—intracellular protein transport, GO:0007264—small GTPase mediated signal transduction, GO:0016192—vesicle‐mediated transport, GO:0016787—hydrolase activity	Potri.002G191400.1, AT1G10630.1	XP_024995498.1, ADP‐ribosylation factor 2 isoform X1 [*Cynara cardunculus* var. *scolymus*]
	MA_31029g0010	N/A	Potri.014G151800.1, AT3G59340.1	XP_023873354.1, solute carrier family 35 member F2 [*Quercus suber*]
	MA_10428833g0010	N/A	N/A	ABR18169.1, unknown [*Picea sitchensis*]
	MA_10428833g0020	GO:0004842—ubiquitin‐protein ligase activity, GO:0005634—nucleus, GO:0006817—phosphate ion transport, GO:0009626—plant‐type hypersensitive response, GO:0009627—systemic acquired resistance, GO:0009697—salicylic acid biosynthetic process, GO:0009751—response to salicylic acid stimulus, GO:0010167—response to nitrate, GO:0010337—regulation of salicylic acid metabolic process, GO:0016036—cellular response to phosphate starvation, GO:0046872—metal ion binding, GO:0080021—response to benzoic acid stimulusGO:0042742—defence response to bacterium	Potri.016G064600.1, AT1G02860.1	ABR18169.1, unknown [*Picea sitchensis*]
PC4	MA_19950g0010	N/A	Potri.014G027200.1	XP_006826575.1, protein ROOT INITIATION DEFECTIVE 3 isoform X2 [*Amborella trichopoda*]
	MA_10433886g0010	GO:0005634—nucleus, GO:0005739—mitochondrion, GO:0005829—cytosol, GO:0007067—mitosis, GO:0009507—chloroplast	Potri.006G263300.2, AT5G47690.3	XP_003635522.2, PREDICTED: sister chromatid cohesion protein PDS5 homolog A‐like [*Vitis vinifera*]
PC5	MA_208236g0010	N/A	Potri.008G210200.1, AT2G47820.2	XP_020082821, uncharacterized protein LOC109706424 isoform X2 [*Ananas comosus*]
*Lirula macrospora*	MA_97571g0020	N/A	N/A	XP_006350818, PREDICTED: acyl‐coenzyme A thioesterase 13‐like [*Solanum tuberosum*]
	MA_97571g0010	GO:0003863—3‐methyl‐2‐oxobutanoate dehydrogenase (2‐methylpropanoyl‐transferring) activity, GO:0005739—mitochondrion, GO:0008152—metabolic process	Potri.005G185400.1, AT5G09300.2	XP_020524476.1, 2‐oxoisovalerate dehydrogenase subunit alpha 2, mitochondrial isoform X2 [*Amborella trichopoda*]
	MA_10432519g0010	N/A	Potri.001G208700.1, AT3G54630.1	XP_021663255.1, kinetochore protein ndc80 [*Hevea brasiliensis*]
***Thekopsora areolata***	MA_10g0010	GO:0008152—metabolic process, GO:0016491—oxidoreductase activity, GO:0016772—transferase activity, transferring phosphorus‐containing groups, GO:0046872—metal ion binding	Potri.007G074700.1, AT5G47000.1	XP_006847800.1, peroxidase 31 [*Amborella trichopoda*]

a The trait upon which the candidate gene associates; PC1–PC6 indicate the associations with loadings on the respective PC; and *L. macrospora* and *T. areolata* indicate associations with the presence/absence data of these fungi among the samples.

b GO terms associated with the gene model in the Norway spruce v1.0 genome portal.

c Angiosperm orthologues to the Norway spruce gene models reported in the Norway spruce v1.0 genome portal.

d The best blastx hit recovered when querying the NCBI nonredundant protein database with the Norway spruce gene model (E‐value cut‐off E < 1.0E‐10).

The candidate gene MA_10433886g0010 associated with PC4 is linked with the GO‐term GO:0007067 (mitosis) in the *P. abies* exAtlas (Nystedt et al., 2013) and its peak expression is found in flushing vegetative shoots although it is also expressed in, for example, buds. MA_19950g0010, the other candidate gene associated with PC4, appears to act in signal perception or transduction as its *Populus* orthologue and best blastx hits both encode WD‐40 repeat family proteins (Table 3). This gene model has a contrasting expression pattern between early‐ and late‐season buds (Figure 2). The gene models associated with PC3 have very low levels of expression in vegetative shoots that have just began to flush (Z4006TR02), while the gene models associated with PC4 and PC5 show relatively high expression in this tissue (Figure 2).

The presumed latent pathogen *S. polyspora*, the cherry spruce rust fungi (*T. areolata*) and the species causing needle cast diseases (*L. macrospora* and *R. kalkhoffii*; Horst, 2013; Kaitera, 2013; Pan et al., 2018; Rajala et al., 2013) met the criteria for targeted GWAS of specific, latent, pathogens. Trait‐association mapping with presence/absence or relative abundances of *S. polyspora* or *R. kalkhoffii* yielded no QTLs, whereas two QTLs for *L. macrospora* presence/absence in the host phyllospere communities and one for *T. areolata* presence/absence were identified (Table 2). None of these QTLs was shared with the QTLs identified with the 1% most abundant OTUs; these traits were associated on independent SNPs, and appeared to be located at different positions in the genome (Table S4). The *L. macrospora* presence/absence in the host phyllosphere communities was associated with SNPs positioned in or adjacent to the Norway spruce gene models MA_97571g0020 and MA_10432519g0010 and the gene model MA_10g0010 was associated with *T. areolata* presence/absence (Table 3). No functional information is associated with MA_97571g0020 in the Norway spruce genome portal, but the PFAM ID PF03801 (HEC/Ndc80p family) is associated with MA_10432519g0010; high relative expression levels of this gene model are found in young vegetative shoots (Z4006TR01) and in immature male cones in the *P. abies* exAtlas (Figure 2). MA_10g0010, associated with *T. areolata,* encodes a previously undescribed class III peroxidase. This candidate gene has a very distinct expression pattern, high expression levels of this gene model are found in young vegetative shoots, developing buds, wood and girdled twigs (Figure 2), and very high relative expression is found in immature female cones (Figure 2).

## 4 DISCUSSION

Foliar fungi of conifers are commonly predicted to be horizontally transmitted and the phyllosphere community of a given tree would be expected to represent a sample of the fungi present in the environment (Millberg et al., 2015; Rodriguez et al., 2009; Terhonen et al., 2019). In angiosperm tree species, the genetic distance between host tree individuals may have a critical impact on assembly of the foliar fungal community (Ahlholm, Helander, Henriksson, Metzler, & Saikkonen, 2002; Cordier et al., 2012). The impact of the tree genotype is unclear in conifers where some studies report an effect of the host genotype on community composition (Rajala et al., 2013), and others find no such correlation (Eusemann et al., 2016). In this study, combining ITS sequencing and GWAS techniques, we provide further evidence that host genotype influences the composition of the phyllosphere community in the significant marker trait‐association mapping of the variation in communities associated with dormant Norway spruce buds.

To our knowledge, no studies of conifer phyllosphere communities have used vegetative buds as a template. However, in a transcriptomic study of various Norway spruce tissues the highest frequency of fungal transcripts was found in needle and bud samples (Delhomme et al., 2015), suggesting that dormant vegetative buds, like needles, may be colonized by phyllosphere fungi. The results from our study further support this, as the taxonomic identities of many of the most heavily sequenced OTUs show affinity to previously reported members of the Norway spruce phyllosphere fungal community. For instance, *Phialophora sessilis*, *Taphrina carpini*, *Ceramothyrium* sp., *Phoma herbarum*, *Aureobasidium pullulans*, *Sydowia polyspora*, *Rhizosphaera kalkhoffii* and *Lirula macrospora* are species which have been reported from studies on Norway spruce needle communities (Menkis et al., 2015; Nguyen et al., 2016; Rajala et al., 2013, 2014). The frequencies and abundances of phyllosphere fungi differ depending on the species present in the local environment (Nguyen et al., 2016; Rodriguez et al., 2009), physiological status of the host tree (Menkis et al., 2015; Rajala et al., 2014), tissue sampled and sample handling. The current study is an illustration of this, as the fraction of yeast‐like fungi was higher here compared to earlier studies. The presence of a large fraction of yeast species may be due to the use of nonsurface‐sterilized explants for DNA extraction, unlike in some previous studies (Nguyen et al., 2016; Rajala et al., 2013). This indicates a need for a similar marker trait association approach with both surface sterilized and nonsterilized explants and perhaps with the amplification and sequencing of the ITS2 region from several single buds from each tree instead of from a pooled sample. However, extraction and community sequencing of nonsurface‐sterilized material as in our study, and the study by Menkis et al. (2015), indicates that the Norway spruce phyllosphere may have quite abundant, yet undescribed and possibly specific, epiphytes as illustrated by OTU_4. This unidentified taxon was one of the most common fungi in the phyllosphere community in the current study and the study by Menkis et al. (2015). It dominated on the first two PCA axes together with the basidiomycete yeast *Curvibasidium cygneicollum*, the common outdoor mould *Cladosporium herbarum,* to some extent the weak pathogen *S. polyspora*, and *Filobasidium wieringae*, suggesting that these axes may be driven primarily by highly frequent and highly abundant phylloplane fungi. It has previously been reported that tree genotypes can influence the occurrence of leaf epiphytes (Bálint et al., 2013; Cordier et al., 2012), but no marker‐trait associations were detected with the two first PCs in this study. Possibly a much larger number of trees would be needed to pick up any association with the community on dormant buds. Other PCs were driven more strongly by well‐known needle and wood colonizers such as *S. polyspora*, the biotrophic cherry spruce rust fungus *Thekopsora areolata* (primarily PC3 and PC4) and *L. macrospora* (PC5 and PC6).

Several, probable latent pathogens were identified among the most abundant species: *T. areolata*, *R. kalkhoffii*, *L. macrospora*, *Botrytis cinerea*, *P. herbarum* and *S. polyspora* (Hennon, 1990; Horst, 2013; Kaitera, 2013; Pan et al., 2018; Rajala et al., 2013). With the exception of *T. areolata*, these species have been reported from Norway spruce phyllosphere communities previously (Menkis et al., 2015; Nguyen et al., 2016; Rajala et al., 2013, 2014). The latent pathogens were, however, much more frequent in our study, found in up to 82% of the samples. It is possible that a surface sterilization treatment would have reduced the frequency of these taxa, but *S. polyspora, L. macrospora, T. areolata* and *R. kalkhoffii* were relatively highly abundant (File S3). Thus, our interpretation is that these four pathogens had colonized asymptomatic buds, acting as latent pathogens.

The interactions between trees and phyllosphere fungi are diverse and not fully understood. It has been reported that endophytic fungi are more diverse and abundant than pathogens within the phyllosphere community (Rodriguez et al., 2009; Terhonen et al., 2019), something that is reflected also in our study. Endophytic fungi in the phyllosphere have the potential to both enhance and reduce tree growth and fitness through various mechanisms (Rodriguez et al., 2009; Terhonen et al., 2019), one of them being the capacity of phyllosphere pathogens to act as both repressors and enablers of disease (Ridout & Newcombe, 2015). A recent study on *Populus* and *Melampsora* established that the interaction of *Melampsora* with endophytic disease repressors is governed by hierarchical contingency rules that determine when and where antagonists in the fungal community reduce plant disease severity, but that this interaction is forestalled by genetic variation for *Melampsora* resistance in *Populus* (Busby, Crutsinger, Barbour, & Newcombe, 2019). If this is a general rule in the interaction between phyllosphere endophytes, pathogens and their hosts, identifying host genetic variation that restricts the colonization by pathogenic fungi or covariates with particular phyllosphere endophyte communities may help to increase our understanding of the interaction between trees and their phyllosphere fungal communities. In this study we were successful in identifying nine such QTLs, highlighting various potential mechanisms.

The gene models MA_10428833g0010 and MA_10428833g0020, associated with PC3, appear to either belong to the same gene as indicated by the shared best blastx hit, ABR18169.1, or possibly represent a tandem duplication of Ubiquitin E3 ligase genes. Angiosperm orthologues of ABR18169.1 have been shown to be linchpins in the responses to nitrate and phosphate starvation (Park, Seo, & Chua, 2014; Peng et al., 2008) and to regulate plant immune responses (Yaeno & Iba, 2008). The potential connection to nitrate and phosphate starvation responses makes this a highly interesting locus as the nutrient status of *Quercus macrocarpa* and *Pinus monticola* leaves is one of the factors that influence their phyllosphere fungal community (Jumpponen & Jones, 2010; Larkin, Hunt, & Ramsey, 2012). Furthermore, a recent study has shown that an impaired phosphate accumulation capacity in *Arabidopsis* led to a strong shift in the phyllosphere fungal community (Finkel et al., 2019).

Several of the potential orthologues of the Norway spruce candidate genes associated with PC3 and PC4 indicate that shoot development and morphogenesis are important factors in shaping genotype‐specific phyllosphere communities in this study. The poplar orthologue of MA_19950g0010, Potri.014G027200.1, encodes a WD‐40 repeat family protein with similarity to *ROOT INITIATION DEFECTIVE 3* (*RID3*). *RID3* has been functionally characterized and the *RID3* locus controls cell division and shoot apical meristem formation in *Arabidopsis* (Tamaki et al., 2009), acting as a negative regulator of key genes. In this context it is noteworthy that the expression of MA_19950g0010 is reported in the *P. abies* exAtlas to be very low during the early phases of Norway spruce bud development just after growth termination when the buds go through a phase of rapid needle initiation and changes in starch and tannin distribution patterns (Hejnowicz & Obarska, 1995). The sister chromatid cohesion proteins PDS5s, the *Arabidopsis* orthologues of MA_104333886g0010 which is associated with PC4, are involved in mitosis, and depletion of this protein lead to severe effects on development, among other traits (Pradillo et al., 2015). Notably, MA_104333886g0010 is most highly expressed in the early phases of bud flush, which is characterized by high mitotic activity and also by changes in both morphology and starch and tannin distribution (Hejnowicz & Obarska, 1995). Interestingly, the candidate genes associated with PC3 show a concomitant downregulation in vegetative shoots that have just began to flush (Z4006TR02), compared to earlier and later phases of shoot development; the genes are differentially expressed between samples taken from needles and stem, indicating that the cellular processes the candidate genes are associated with are not ubiquitous in the phyllosphere and that their absence could be important for colonization success for certain fungal taxa. The observations associated with PC3 and PC4 suggest that bud flush, and the changes in distribution of energy and defensive metabolites that the tissue transitions through, may be a critical phase in the colonization of Norway spruce by the studied phyllosphere fungal community and their later occurrence in dormant buds. This is in line with the work by Eusemann et al. (2016) who postulated that tree phenology was one of the drivers between assembly of the phyllosphere community in their study. Phenology (timing of bud burst) is under very strong genetic control in Norway spruce, and it has a narrow sense heritability of approximately 0.8 (Hannerz, Sonesson, & Ekberg, 1999). The function and expression patterns of each of the identified Norway spruce candidate genes, and perhaps the role of phenology, in the interaction between phyllosphere fungi and Norway spruce, will need to be tested in future experiments.

One of the candidate genes associated with the presence/absence of *L. macrospora* in the communities*,* MA_10432519g0010, has similarity to the HEC/Ndc80p family proteins. These proteins are part of the kinetochore complex, which provides an attachment site for spindle fibres in the centromere of chromosomes, and thus are important for cell division (Shin, Jeong, Park, Kim, & Lee, 2018). Disruption of the kinetochore complex may affect, for example, morphogenesis (Du & Dawe, 2007; Lermontova et al., 2011; Shin et al., 2018). It may be difficult to reconcile a potential role of MA_10432519g0010 in development and morphogenesis with control of *L. macrospora* colonization as disease symptoms are commonly seen on second‐year needles (Butin, 1995). However, it has been suggested that *L. macrospora* infects flushing tissues in the spring with a latency period of about 1 year until symptoms are visible (Hennon, 1990), which would connect the peak transcriptional activity of the candidate gene with the crucial infection phase.

MA_10g0010, which harbours an SNP associated with the presence of *T. areolata* in the phyllosphere fungal community is a previously undescribed member of the class III peroxidase family. The gene model shows an expression pattern strongly associated with differentiating and lignifying tissues in the *P. abies* exAtlas, particularly with young female cones. Clearly the candidate gene is active in tissues susceptible to the pathogen, and basidiospores of *T. areolata* are thought to infect Norway spruce cones and young shoots in the spring (Hietala, Solheim, & Fossdal, 2007; Kuporevich & Transhel, 1957). Thus, it is not far‐fetched to imagine that MA_10g0010 may be associated with processes in these tissues that control their vulnerability to colonization, possibly through cell‐wall enforcement (Elfstrand et al., 2001; Fagerstedt, Kukkola, Koistinen, Takahashi, & Marjamaa, 2010; Kärkönen, Warinowski, Teeri, Simola, & Fry, 2009; Marjamaa et al., 2006) or processes controlling the timing of bud break and bud flush; overexpression of the class III peroxidase *SPI2* in Norway spruce plants led to significant delays of these process compared to in wild‐type Norway spruce plants (Clapham, Häggman, Elfstrand, Aronen, & Arnold, 2003; Elfstrand et al., 2001).

Combining molecular identification and barcoding of phyllosphere fungi with GWAS provides insight into host‐encoded factors affecting the assembly of phyllosphere communities and the colonization of needle pathogens. Taken together, GWAS results suggest that processes in the morphogenesis and flush of the Norway spruce shoot exert a strong influence on the dominant players in the phyllosphere community detected in dormant buds.

## AUTHOR CONTRIBUTIONS

Conception of the study: Å.O., J.S., M.E., M.R.G.G., K.L., B.K. and H.X.W.; planning the study: M.E., Å.O., J.B., L.Z., M.R.G.G., K.L. and J.S.; execution of the study: M.E., J.B., L.Z. and M.R.G.G.; drafting the manuscript: M.E., J.B. and L.Z.; all authors read and commented on the text. M.E. wrote the final manuscript, and all authors read and approved the final version.

## Data Availability

ITS7/ITS4 raw read data are stored at the NCBI Sequence Read Archive in the Bioproject PRJNA590990. Contact M.Rosario.Garcia@slu.se for genotype information

## References

[mec15314-bib-0001] Ahlholm, J. U. , Helander, M. , Henriksson, J. , Metzler, M. , & Saikkonen, K. (2002). Environmental conditions and host genotype direct genetic diversity of *Venturia ditricha,* a fungal endophyte of birch trees. Evolution, 56, 1566–1573. 10.1111/j.0014-3820.2002.tb01468.x 12353749

[mec15314-bib-0002] Arnold, A. E. , Henk, D. A. , Eells, R. L. , Lutzoni, F. , & Vilgalys, R. (2007). Diversity and phylogenetic affinities of foliar fungal endophytes in loblolly pine inferred by culturing and environmental PCR. Mycologia, 99, 185–206. 10.1080/15572536.2007.11832578 17682771

[mec15314-bib-0003] Baison, J. , Vidalis, A. , Zhou, L. , Chen, Z.‐Q. , Li, Z. , Sillanpää, M. J. , … García‐Gil, M. R. (2019). Genome‐Wide Association Study (GWAS) identified novel candidate loci affecting wood formation in Norway spruce. The Plant Journal, 100, 83–100. 10.1111/tpj.14429 31166032 PMC6852177

[mec15314-bib-0004] Bálint, M. , Tiffin, P. , Hallström, B. , O’Hara, R. B. , Olson, M. S. , Fankhauser, J. D. , … Schmitt, I. (2013). Host genotype shapes the foliar fungal microbiome of balsam poplar (*Populus balsamifera*). PLoS ONE, 8, e53987 10.1371/journal.pone.0053987 23326555 PMC3543377

[mec15314-bib-0005] Bernhardsson, C. , Vidalis, A. , Wang, X. , Scofield, D. G. , Schiffthaler, B. , Baison, J. , … Ingvarsson, P. K. (2019). An ultra‐dense haploid genetic map for evaluating the highly fragmented genome assembly of Norway spruce (*Picea abies*). G3: Genes|genomes|genetics, 9, 1623–1632. 10.1534/g3.118.200840 30898899 PMC6505157

[mec15314-bib-0006] Busby, P. E. , Crutsinger, G. , Barbour, M. , & Newcombe, G. (2019). Contingency rules for pathogen competition and antagonism in a genetically based, plant defense hierarchy. Ecology and Evolution, 9, 6860–6868. 10.1002/ece3.5253 31380021 PMC6662256

[mec15314-bib-0007] Butin, H. H. (1995). Tree diseases and disorders: Causes, biology, and control in forest and amenity trees. Oxford: Oxford University Press.

[mec15314-bib-0008] Cingolani, P. , Platts, A. , Wang, L. L. , Coon, M. , Nguyen, T. , Wang, L. , … Ruden, D. M. (2012). A program for annotating and predicting the effects of single nucleotide polymorphisms, SnpEff. Fly, 6, 80–92. 10.4161/fly.19695 22728672 PMC3679285

[mec15314-bib-0009] Clapham, D. H. , Häggman, H. , Elfstrand, M. , Aronen, T. , & von Arnold, S. (2003). Transformation of Norway spruce (*Picea abies*) by particle bombardment In Jackson J. F. & Linskens H. F. (Eds.), Genetic transformation of plants (pp. 127–146). Berlin: Springer.

[mec15314-bib-0010] Clemmensen, K. E. , Bahr, A. , Ovaskainen, O. , Dahlberg, A. , Ekblad, A. , Wallander, H. , … Lindahl, B. D. (2013). Roots and associated fungi drive long‐term carbon sequestration in boreal forest. Science, 339, 1615–1618. 10.1126/science.1231923 23539604

[mec15314-bib-0011] Clemmensen, K. E. , Ihrmark, K. , Durling, M. B. , & Lindahl, B. D. (2016). Sample preparation for fungal community analysis by high‐throughput sequencing of barcode amplicons In Martin F. & Uroz S. (Eds.), Microbial environmental genomics (MEG) (pp. 61–88). New York: Springer. 10.1007/978-1-4939-3369-3_4 26791497

[mec15314-bib-0012] Cordier, T. , Robin, C. , Capdevielle, X. , Desprez‐Loustau, M.‐L. , & Vacher, C. (2012). Spatial variability of phyllosphere fungal assemblages: Genetic distance predominates over geographic distance in a European beech stand (*Fagus sylvatica*). Fungal Ecology, 5, 509–520. 10.1016/j.funeco.2011.12.004

[mec15314-bib-0013] Delhomme, N. , Sundström, G. , Zamani, N. , Lantz, H. , Lin, Y.‐C. , Hvidsten, T. R. , … Street, N. R. (2015). Serendipitous meta‐transcriptomics: The fungal community of Norway spruce (*Picea abies*). PLoS ONE, 10, e0139080 10.1371/journal.pone.0139080 26413905 PMC4586145

[mec15314-bib-0014] DePristo, M. A. , Banks, E. , Poplin, R. , Garimella, K. V. , Maguire, J. R. , Hartl, C. , … Daly, M. J. (2011). A framework for variation discovery and genotyping using next‐generation DNA sequencing data. Nature Genetics, 43, 491–498. 10.1038/ng.806 21478889 PMC3083463

[mec15314-bib-0015] Du, Y. , & Dawe, R. K. (2007). Maize NDC80 is a constitutive feature of the central kinetochore. Chromosome Research, 15, 767–775. 10.1007/s10577-007-1160-z 17643192

[mec15314-bib-0016] Elfstrand, M. , Fossdal, C. , Sitbon, F. , Olsson, O. , Lönneborg, A. , & von Arnold, S. (2001). Overexpression of the endogenous peroxidase‐like gene spi 2 in transgenic Norway spruce plants results in increased total peroxidase activity and reduced growth. Plant Cell Reports, 20, 596–603. 10.1007/s002990100360

[mec15314-bib-0017] Eusemann, P. , Schnittler, M. , Nilsson, R. H. , Jumpponen, A. , Dahl, M. B. , Würth, D. G. , … Unterseher, M. (2016). Habitat conditions and phenological tree traits overrule the influence of tree genotype in the needle mycobiome–*Picea glauca* system at an arctic treeline ecotone. New Phytologist, 211, 1221–1231. 10.1111/nph.13988 27144386

[mec15314-bib-0018] Fagerstedt, K. V. , Kukkola, E. M. , Koistinen, V. V. T. , Takahashi, J. , & Marjamaa, K. (2010). Cell wall lignin is polymerised by class iii secretable plant peroxidases in Norway spruce. Journal of Integrative Plant Biology, 52, 186–194. 10.1111/j.1744-7909.2010.00928.x 20377680

[mec15314-bib-0019] Farjon, A. , & Page, C. N. (1999). Conifers. Status survey and conservation action plan. Gland, Switzerland: IUCN.

[mec15314-bib-0020] Finkel, O. M. , Salas‐González, I. , Castrillo, G. , Spaepen, S. , Law, T. F. , Jones, C. D. , & Dangl, J. L. (2019). The effects of soil phosphorous content on microbiota are driven by the plant phosphate starvation response. bioRxiv, 608133 10.1101/608133 PMC6876890 31721759

[mec15314-bib-0021] Gao, H. , Wu, Y. , Li, J. , Li, H. , Li, J. , & Yang, R. (2014). Forward LASSO analysis for high‐order interactions in genome‐wide association study. Briefings in Bioinformatics, 15, 552–561. 10.1093/bib/bbt037 23775311

[mec15314-bib-0022] Hammer, O. , Harper, D. A. T. , & Ryan, P. D. (2001). PAST: Paleontological statistics software package for education and data analysis. Palaeontologia Electronica, 4, 1–9.

[mec15314-bib-0023] Hannerz, M. , Sonesson, J. , & Ekberg, I. (1999). Genetic correlations between growth and growth rhythm observed in a short‐term test and performance in long‐term field trials of Norway spruce. Canadian Journal of Forest Research, 29, 768–778. 10.1139/x99-056

[mec15314-bib-0024] Hanso, M. , & Drenkhan, R. (2012). *Lophodermium* needle cast, insect defoliation and growth responses of young Scots pines in Estonia. Forest Pathology, 42, 124–135. 10.1111/j.1439-0329.2011.00728.x

[mec15314-bib-0025] Hejnowicz, A. , & Obarska, E. (1995). Structure and development of vegetative buds, from the lower crown of *Picea abies* . Annals of Forest Science, 52, 433–447.

[mec15314-bib-0026] Hennon, P. E. (1990). Sporulation of *Lirula macrospora* and symptom development on Sitka spruce in southeast Alaska. Plant Disease, 74, 316–319. 10.1094/PD-74-0316

[mec15314-bib-0027] Hietala, A. M. , Solheim, H. , & Fossdal, C. G. (2007). Real‐time PCR‐based monitoring of DNA pools in the tri‐trophic interaction between Norway spruce, the rust *Thekopsora areolata*, and an opportunistic ascomycetous *Phomopsis* sp. Phytopathology, 98, 51–58. 10.1094/PHYTO-98-1-0051 18943238

[mec15314-bib-0028] Horst, R. K. (2013). Needle casts In Horst R. K. (Ed.), Westcott's plant disease handbook (pp. 245–249). Dordrecht: Springer.

[mec15314-bib-0029] Horton, M. W. , Bodenhausen, N. , Beilsmith, K. , Meng, D. , Muegge, B. D. , Subramanian, S. , … Bergelson, J. (2014). Genome‐wide association study of *Arabidopsis thaliana* leaf microbial community. Nature Communications, 5, 5320 10.1038/ncomms6320 PMC4232226 25382143

[mec15314-bib-0030] Ihrmark, K. , Bödeker, I. T. M. , Cruz‐Martinez, K. , Friberg, H. , Kubartova, A. , Schenck, J. , … Lindahl, B. D. (2012). New primers to amplify the fungal ITS2 region‐evaluation by 454‐sequencing of artificial and natural communities. FEMS Microbiology Ecology, 82, 666–677. 10.1111/j.1574-6941.2012.01437.x 22738186

[mec15314-bib-0031] Jumpponen, A. , & Jones, K. L. (2010). Seasonally dynamic fungal communities in the *Quercus macrocarpa* phyllosphere differ between urban and nonurban environments. New Phytologist, 186, 496–513. 10.1111/j.1469-8137.2010.03197.x 20180911

[mec15314-bib-0032] Kaitera, J. (2013). *Thekopsora* and *Chrysomyxa* cone rusts damage Norway spruce cones after a good cone crop in Finland. Scandinavian Journal of Forest Research, 28, 217–222.

[mec15314-bib-0033] Kärkönen, A. , Warinowski, T. , Teeri, T. H. , Simola, L. K. , & Fry, S. C. (2009). On the mechanism of apoplastic H_2_O_2_ production during lignin formation and elicitation in cultured spruce cells—Peroxidases after elicitation. Planta, 230, 553–567. 10.1007/s00425-009-0968-5 19544069

[mec15314-bib-0034] Kubartová, A. , Ottosson, E. , Dahlberg, A. , & Stenlid, J. (2012). Patterns of fungal communities among and within decaying logs, revealed by 454 sequencing. Molecular Ecology, 21, 4514–4532. 10.1111/j.1365-294X.2012.05723.x 22882383

[mec15314-bib-0035] Kuporevich, V. , & Transhel, V. (1957). Cryptogamic plants of the USSR: Rust fungi. Jerusalem: Keter Press.

[mec15314-bib-0036] Larkin, B. G. , Hunt, L. S. , & Ramsey, P. W. (2012). Foliar nutrients shape fungal endophyte communities in Western white pine (*Pinus monticola*) with implications for white‐tailed deer herbivory. Fungal Ecology, 5, 252–260. 10.1016/j.funeco.2011.11.002

[mec15314-bib-0037] Lermontova, I. , Koroleva, O. , Rutten, T. , Fuchs, J. , Schubert, V. , Moraes, I. , … Schubert, I. (2011). Knockdown of CENH3 in *Arabidopsis* reduces mitotic divisions and causes sterility by disturbed meiotic chromosome segregation. The Plant Journal, 68, 40–50. 10.1111/j.1365-313X.2011.04664.x 21635586

[mec15314-bib-0038] Li, H. , & Durbin, R. (2010). Fast and accurate long‐read alignment with Burrows‐Wheeler transform. Bioinformatics, 26, 589–595. 10.1093/bioinformatics/btp698 20080505 PMC2828108

[mec15314-bib-0039] Li, Z. , Hallingbäck, H. R. , Abrahamsson, S. , Fries, A. , Andersson Gull, B. , Sillanpää, M. J. , & García‐Gil, M. R. (2014). Functional multi‐locus QTL mapping of temporal trends in Scots pine wood traits. G3: Genes|Genomes|Genetics, 4(12), 2365–2379. 10.1534/g3.114.014068 25305041 PMC4267932

[mec15314-bib-0040] Li, Z. , & Sillanpää, M. J. (2015). Dynamic quantitative trait locus analysis of plant phenomic data. Trends in Plant Science, 20, 822–833. 10.1016/j.tplants.2015.08.012 26482958

[mec15314-bib-0041] Marjamaa, K. , Hildén, K. , Kukkola, E. , Lehtonen, M. , Holkeri, H. , Haapaniemi, P. , … Lundell, T. (2006). Cloning, characterization and localization of three novel class III peroxidases in lignifying xylem of Norway spruce (*Picea abies*). Plant Molecular Biology, 61, 719–732. 10.1007/s11103-006-0043-6 16897487

[mec15314-bib-0042] McKenna, A. , Hanna, M. , Banks, E. , Sivachenko, A. , Cibulskis, K. , Kernytsky, A. , … DePristo, M. A. (2010). The genome analysis toolkit: A MapReduce framework for analyzing next‐generation DNA sequencing data. Genome Research, 20, 1297–1303. 10.1101/gr.107524.110 20644199 PMC2928508

[mec15314-bib-0043] Meinshausen, N. , & Bühlmann, P. (2010). Stability selection. Journal of the Royal Statistical Society: Series B (Statistical Methodology), 72, 417–473. 10.1111/j.1467-9868.2010.00740.x

[mec15314-bib-0044] Menkis, A. , Marciulynas, A. , Gedminas, A. , Lynikiene, J. , & Povilaitiene, A. (2015). High‐throughput sequencing reveals drastic changes in fungal communities in the phyllosphere of Norway spruce (*Picea abies*) following invasion of the spruce bud scale (*Physokermes piceae*). Microbial Ecology, 70, 904–911. 10.1007/s00248-015-0638-z 26054703

[mec15314-bib-0045] Millberg, H. , Boberg, J. , & Stenlid, J. (2015). Changes in fungal community of Scots pine (*Pinus sylvestris*) needles along a latitudinal gradient in Sweden. Fungal Ecology, 17, 126–139. 10.1016/j.funeco.2015.05.012

[mec15314-bib-0046] Moler, E. R. V. , & Aho, K. (2018). Whitebark pine foliar fungal endophyte communities in the southern Cascade Range, USA: Host mycobiomes and white pine blister rust. Fungal Ecology, 33, 104–114. 10.1016/j.funeco.2018.02.003

[mec15314-bib-0047] Nguyen, D. , Boberg, J. , Ihrmark, K. , Stenström, E. , & Stenlid, J. (2016). Do foliar fungal communities of Norway spruce shift along a tree species diversity gradient in mature European forests? Fungal Ecology, 23, 97–108. 10.1016/j.funeco.2016.07.003

[mec15314-bib-0048] Nystedt, B. , Street, N. R. , Wetterbom, A. , Zuccolo, A. , Lin, Y.‐C. , Scofield, D. G. , … Jansson, S. (2013). The Norway spruce genome sequence and conifer genome evolution. Nature, 497, 579–584. 10.1038/nature12211 23698360

[mec15314-bib-0049] Pan, Y. , Ye, H. , Lu, J. , Chen, P. , Zhou, X.‐.‐D. , Qiao, M. , … Yu, Z.‐.‐F. (2018). Isolation and identification of *Sydowia polyspora* and its pathogenicity on *Pinus yunnanensis* in Southwestern China. Journal of Phytopathology, 166, 386–395.

[mec15314-bib-0050] Park, B. S. , Seo, J. S. , & Chua, N.‐H. (2014). NITROGEN LIMITATION ADAPTATION Recruits PHOSPHATE2 to target the phosphate transporter PT2 for degradation during the regulation of *Arabidopsis* phosphate homeostasis. The Plant Cell, 26, 454–464. 24474629 10.1105/tpc.113.120311 PMC3963589

[mec15314-bib-0051] Peng, M. , Hudson, D. , Schofield, A. , Tsao, R. , Yang, R. , Gu, H. , … Rothstein, S. J. (2008). Adaptation of *Arabidopsis* to nitrogen limitation involves induction of anthocyanin synthesis which is controlled by the *NLA* gene. Journal of Experimental Botany, 59, 2933–2944. 10.1093/jxb/ern148 18552353 PMC2504352

[mec15314-bib-0052] Pradillo, M. , Knoll, A. , Oliver, C. , Varas, J. , Corredor, E. , Puchta, H. , & Santos, J. L. (2015). Involvement of the cohesin cofactor PDS5 (SPO76) during meiosis and DNA repair in *Arabidopsis thaliana* . Frontiers Plant Science, 6 10.3389/fpls.2015.01034 PMC4664637 26648949

[mec15314-bib-0053] Prunier, J. , Verta, J.‐P. , & MacKay, J. J. (2016). Conifer genomics and adaptation: At the crossroads of genetic diversity and genome function. New Phytologist, 209, 44–62. 10.1111/nph.13565 26206592

[mec15314-bib-0054] Rajala, T. , Velmala, S. M. , Tuomivirta, T. , Haapanen, M. , Müller, M. , & Pennanen, T. (2013). Endophyte communities vary in the needles of Norway spruce clones. Fungal Biology, 117, 182–190. 10.1016/j.funbio.2013.01.006 23537875

[mec15314-bib-0055] Rajala, T. , Velmala, S. M. , Vesala, R. , Smolander, A. , & Pennanen, T. (2014). The community of needle endophytes reflects the current physiological state of Norway spruce. Fungal Biology, 118, 309–315. 10.1016/j.funbio.2014.01.002 24607354

[mec15314-bib-0056] Ridout, M. , & Newcombe, G. (2015). The frequency of modification of *Dothistroma* pine needle blight severity by fungi within the native range. Forest Ecology and Management, 337, 153–160. 10.1016/j.foreco.2014.11.010

[mec15314-bib-0057] Rodriguez, R. J. , White, J. F. Jr , Arnold, A. E. , & Redman, R. S. (2009). Fungal endophytes: Diversity and functional roles. New Phytologist, 182, 314–330. 10.1111/j.1469-8137.2009.02773.x 19236579

[mec15314-bib-0058] Roman‐Reyna, V. , Pinili, D. , Borjaa, F. N. , Quibod, I. , Groen, S. C. , Mulyaningsih, E. S. , … Oliva, R. (2019). The rice leaf microbiome has a conserved community structure controlled by complex host‐microbe interactions. bioRxiv, 615278

[mec15314-bib-0059] Rosling, A. , Cox, F. , Cruz‐Martinez, K. , Ihrmark, K. , Grelet, G.‐A. , Lindahl, B. D. , … James, T. Y. (2011). Archaeorhizomycetes: Unearthing an ancient class of ubiquitous soil fungi. Science, 333, 876–879. 10.1126/science.1206958 21836015

[mec15314-bib-0060] Saikkonen, K. (2007). Forest structure and fungal endophytes. Fungal Biology Reviews, 21, 67–74. 10.1016/j.fbr.2007.05.001

[mec15314-bib-0061] Savolainen, O. , Pyhäjärvi, T. , & Knürr, T. (2007). Gene flow and local adaptation in trees. Annual Review of Ecology, Evolution, and Systematics, 38, 595–619. 10.1146/annurev.ecolsys.38.091206.095646

[mec15314-bib-0062] Seena, S. , & Monroy, S. (2016). Preliminary insights into the evolutionary relationships of aquatic hyphomycetes and endophytic fungi. Fungal Ecology, 19, 128–134. 10.1016/j.funeco.2015.07.007

[mec15314-bib-0063] Shin, J. , Jeong, G. , Park, J.‐Y. , Kim, H. , & Lee, I. (2018). *MUN* (*MERISTEM UNSTRUCTURED*), encoding a SPC24 homolog of NDC80 kinetochore complex, affects development through cell division in *Arabidopsis thaliana* . The Plant Journal, 93, 977–991. 29356153 10.1111/tpj.13823

[mec15314-bib-0064] Sieber, T. N. (2007). Endophytic fungi in forest trees: Are they mutualists? Fungal Biology Reviews, 21, 75–89. 10.1016/j.fbr.2007.05.004

[mec15314-bib-0065] Tamaki, H. , Konishi, M. , Daimon, Y. , Aida, M. , Tasaka, M. , & Sugiyama, M. (2009). Identification of novel meristem factors involved in shoot regeneration through the analysis of temperature‐sensitive mutants of *Arabidopsis* . The Plant Journal, 57, 1027–1039. 10.1111/j.1365-313X.2008.03750.x 19054368

[mec15314-bib-0066] Team, R . (2015). RStudio: integrated development for R. Boston: RStudio Inc Retrieved from http://www.rstudio.com

[mec15314-bib-0067] Tedersoo, L. , Bahram, M. , Põlme, S. , Kõljalg, U. , Yorou, N. S. , Wijesundera, R. , … Abarenkov, K. (2014). Global diversity and geography of soil fungi. Science, 346(6213), 1256688– 10.1126/science.1256688 25430773

[mec15314-bib-0068] Terhonen, E. , Blumenstein, K. , Kovalchuk, A. , & Asiegbu, F. O. (2019). Forest tree microbiomes and associated fungal endophytes: Functional roles and impact on forest health. Forests, 10, 42 10.3390/f10010042

[mec15314-bib-0069] Vidalis, A. , Scofield, D. G. , Neves, L. G. , Bernhardsson, C. , García‐Gil, M. R. , & Ingvarsson, P. K. (2018). Design and evaluation of a large sequence‐capture probe set and associated SNPs for diploid and haploid samples of Norway spruce (*Picea abies*). bioRxiv, 10.1101/291716

[mec15314-bib-0070] Voříšková, J. , & Baldrian, P. (2012). Fungal community on decomposing leaf litter undergoes rapid successional changes. The ISME Journal, 7, 477 10.1038/ismej.2012.116 23051693 PMC3578564

[mec15314-bib-0071] Wallace, J. G. , Kremling, K. A. , Kovar, L. L. , & Buckler, E. S. (2018). Quantitative genetics of the maize leaf microbiome. Phytobiomes Journal, 2, 208–224. 10.1094/PBIOMES-02-18-0008-R

[mec15314-bib-0072] Yaeno, T. , & Iba, K. (2008). BAH1/NLA, a RING‐type ubiquitin E3 Ligase, regulates the accumulation of salicylic acid and immune responses to *Pseudomonas syringae* DC3000. Plant Physiology, 148, 1032–1041. 18753285 10.1104/pp.108.124529 PMC2556844

[mec15314-bib-0073] Zou, H. (2006). The adaptive lasso and its oracle properties. Journal of the American Statistical Association, 101, 1418–1429.

