# Supplementary material for: Genotypic variation in Norway spruce correlates to fungal communities in vegetative buds
Source: Mol Ecol. 2019 Dec 9;29(1):199–213. doi: 10.1111/mec.15314 (PMC7003977; doi:10.1111/mec.15314)
Supplement: Supplementary file 1 [file MEC-29-199-s001.zip › mec15314-sup-0002-SupinfoS1.pdf]

Supporting information S1: Sequencing depth and inclusion of OTUs in marker-trait associations

In the current study 85% of the reads that passed the quality filtering criteria stemmed from Norway spruce ITS2 (Figure A).

Fig. A

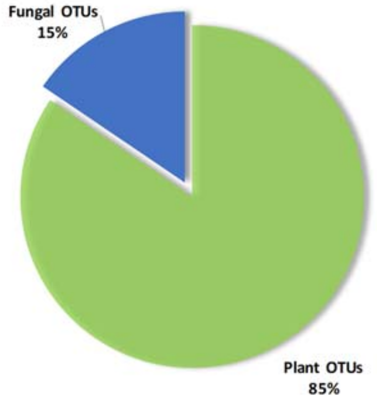

This was an unexpected result since the gITS7/ITS4, normally do not amplify as much as Norway spruce ITS2 when analysing communities in “comparable” Norway spruce tissues such as needles or flushing buds e.g. Nguyen et al (2016) and Menkis et al (2015) both reports non-fungal reads in the order of 13%. Thus the projected average of 1300 reads per sample were expected been more sufficient for the study’s objectives but in retrospect sequencing to a greater depth would probably have been beneficial and allowed us to include more OTUs in the analysis and to reduce uncertainties and noise in the dataset possibly stemming from the relatively low depth.

The unexpectedly low fraction of fungal reads from dormant vegetative buds suggested that dormant vegetative buds may have a smaller ratio of fungal/host DNA than e.g. the surrounding needles. This would be is in agreement with the literature reporting that the number of isolated endophytic strains are greater from older needles eg. (Hata *et al.* 1998). It is however, important to keep in mind that to successfully run the GWAS we needed to reach a balance between the phenotype data and genotype data. Therefore a set strict pre-defined criteria on both OTU size and presence in the samples was applied to the dataset and analysed with multivariate ordination methods before moving ahead with the subsequent analyses (top 1% OTUs, top 2%, top 5%, including 95% of the reads, 90% of the reads etc.). The effect of applying the criteria 1% OTUs, top 2%, top 5%, including 95% of the reads is on the fraction of reads and number of clusters presented in the graph below (Figure B).

Fig. B

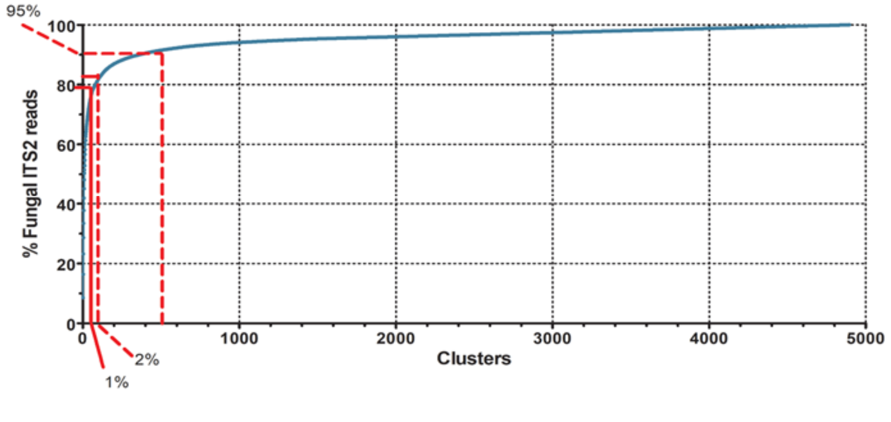

The tests based on these criteria suggested that the most conservative criterion, restricting the analyses to the 1% largest OTU clusters, comprising 80% of the fungal reads, with a median presence

of the OTU in 184 samples (38% of the sample) would mean that each included OTU had some chance of influencing the genetic associations in the subsequent marker-trait association.

Cited literature

Hata K, Futai K, Tsuda M (1998) Seasonal and needle age-dependent changes of the endophytic mycobiota in *Pinus thunbergii* and *Pinus densiflora* needles. *Canadian Journal of Botany* **76**, 245-250.

Menkis A, Marciulynas A, Gedminas A, Lynikiene J, Povilaitiene A (2015) High-Throughput Sequencing Reveals Drastic Changes in Fungal Communities in the Phyllosphere of Norway Spruce (*Picea abies*) Following Invasion of the Spruce Bud Scale (*Physokermes piceae*). *Microbial Ecology* **70**, 904-911.

Nguyen D, Boberg J, Ihrmark K, Stenström E, Stenlid J (2016) Do foliar fungal communities of Norway spruce shift along a tree species diversity gradient in mature European forests? *Fungal Ecology* **23**, 97-108.
