# Supplementary material for: Genotypic variation in Norway spruce correlates to fungal communities in vegetative buds
Source: Mol Ecol. 2019 Dec 9;29(1):199–213. doi: 10.1111/mec.15314 (PMC7003977; doi:10.1111/mec.15314)
Supplement: Supplementary file 1 [file MEC-29-199-s001.zip › mec15314-sup-0004-TableS4.docx]

**Supporting information Table S4**: Position of markers in a dense genetic map of the highly fragmented Norway spruce genome assembly

| **Trait^a^** | **SNP^b^** | **LG^c^** | **Probe^d^** | **cM^e^** |
| --- | --- | --- | --- | --- |
| PC3 | MA_10428833_21190 | 3 | MA_10428833:1 | 84.9 |
|  | MA_24477_24501 | 3 | MA_24477:1 | 111.8 |
|  | MA_31029 _9337 | 9 | MA_31029:1 | 61.4 |
| PC4 | MA_19950_16139 |  | N/A |  |
|  | MA_10433886_12255 |  | N/A |  |
| PC5 | MA_208236_3389 | 5 | MA_208236:1 | 30.1 |
|  |  |  |  |  |
| *L. macrospora* | MA_10432519_8378 | 5 | MA_10432519:1 | 131.2 |
|  | MA_97571_20468 | 9 | MA_97571:2 | 213.1 |
| *T. areolata* | MA_10_25927 | 2 | MA_10:2 | 49.3 |

^a^Trait, specify the trait upon which the marker associate; PC3-5 indicate the associations with loadings on the respective PC; and *L. macrospora* and *T. areolata* specify associations with the presence/absence data of these fungi among the samples ^b^SNP: The SNP name was composed of the contig (MA_number) and SNP position on the contig. For detailed explanation see table 2; ^c^ LG group in which SNPs from the contigs probe(s) are positioned in the genetic map constructed by Bernhardsson et al (2019) feature allelic variation associated with the SNP; ^d^ Probes used by Bernhardsson et al (2019) , and ^e^ position of the marker in the linkage group in centiMorgans (cM).
