# Supplementary figures and images for: Genotypic variation in Norway spruce correlates to fungal communities in vegetative buds
Source: Mol Ecol. 2019 Dec 9;29(1):199–213. doi: 10.1111/mec.15314 (PMC7003977; doi:10.1111/mec.15314)

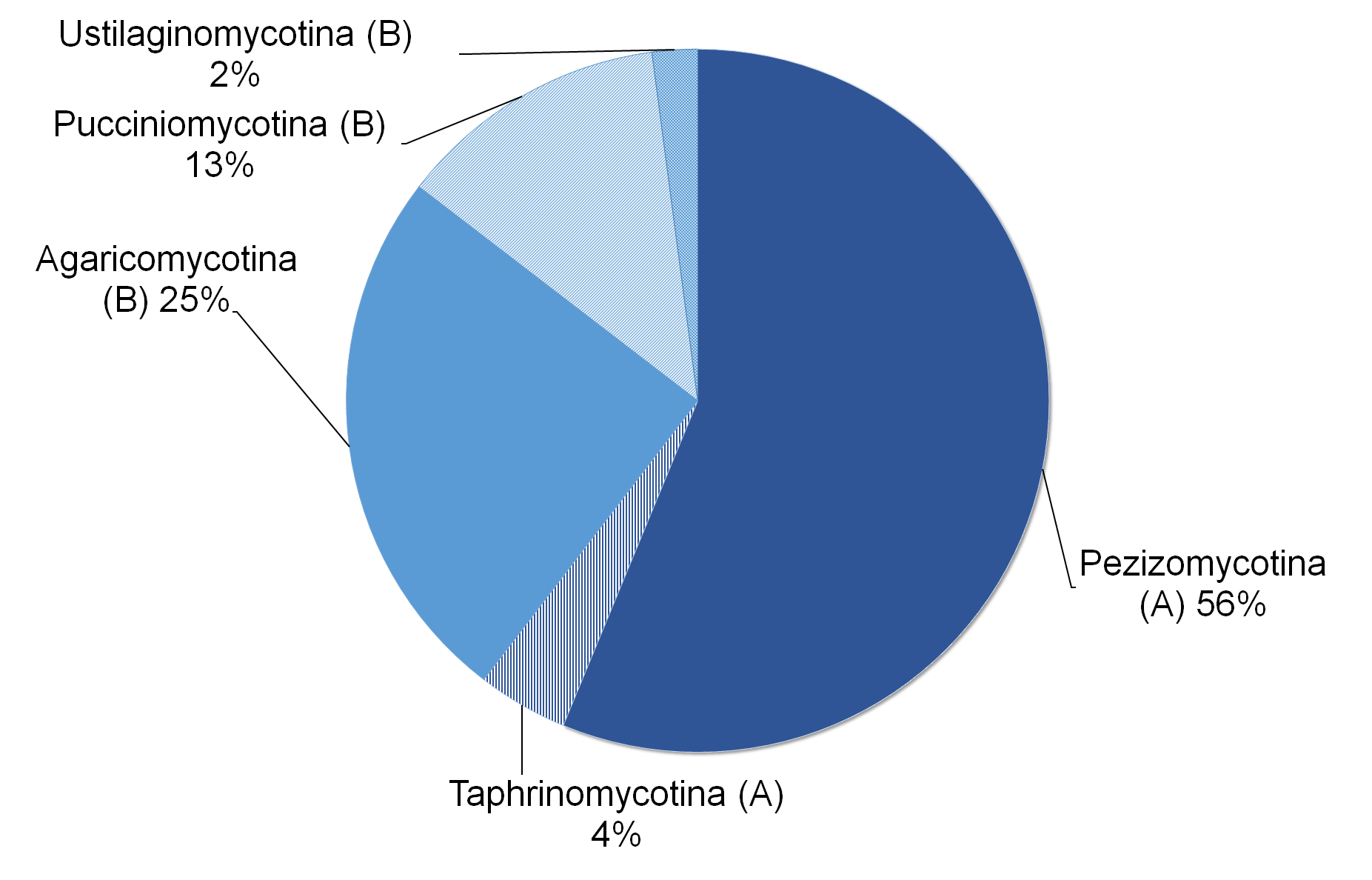

Supplement: Supplementary file 1 [file MEC-29-199-s001.zip › mec15314-sup-0001-FigS2.jpg]
